# The Impact of Air Pollution on Hospitalization for Cardiovascular and Cerebrovascular Disease in Shenyang, China

**DOI:** 10.18502/ijph.v49i8.3891

**Published:** 2020-08

**Authors:** Qingquan REN, Shuyin LI, Chunling XIAO, Jiazhi ZHANG, Hong LIN, Shuai WANG

**Affiliations:** 1. Department of Labor and Environmental Hygiene, Shenyang Medical College, Liaoning 110034, P.R. China; 2. Key Laboratory of Environmental Pollution and Microecology of Liaoning Province, Shenyang Medical College, Shenyang, Liaoning 110034, P.R. China; 3. Department of Pathogenic Biology, Shenyang Medical College, Shenyang, Liaoning 110034, P.R. China; 4. Shenyang Environmental Monitoring Center Station, Shenyang, Liaoning 110016, P.R. China

**Keywords:** Cardiovascular disease, Cerebrovascular disease, Pollutant

## Abstract

**Background:**

The aim of this study was to investigate the overall impact of PM_2.5_, PM_10_, NO_2_, SO_2_, CO, and O_3_ on the admission of cardiovascular and cerebrovascular disease.

**Methods:**

We collected data on cardiovascular and cerebrovascular disease admissions from two hospitals in Shenyang Liaoning, China from Jan 2014 to Dec 2017, as well as daily measurements of six pollutants at 11 sites in Shenyang. The generalized additive model was used to assess the association between daily contaminants and admission to cardiovascular and cerebrovascular disease.

**Results:**

The single-contamination model showed a significant correlation between NO_2_, O_3_, PM_10_ and cardiovascular and cerebrovascular diseases at lag0 day. Air pollutants had lag effects on different gender groups. Excess relative risks (ERs) associated with a 10 μg/m^3^ increase were 1.522(1.057, 1.988) on lag02 for NO_2_, 0.547% (0.367%, 0.728%), 0.133% (0.061%, 0.205%) on lag3 for O_3_ and PM_10_. The dual pollutant model showed that the effects of NO_2_, O_3_, and PM_10_ after adjusting the influence of other pollutants were still statistically significant.

**Conclusion:**

Short-term exposure to ambient air pollution (NO_2_, O_3_, and PM_10_) may be associated with an increased risk of daily cardiovascular and cerebrovascular admission, which may provide reliable evidence for further understanding of the potential adverse effects of air pollution on cardiovascular and cerebrovascular diseases.

## Introduction

Cardiovascular and cerebrovascular diseases are generally referred to cardiac, cerebral ischemic or hemorrhagic diseases caused by various factors such as diabetes, dyslipidemia, hypertension, overweight or obesity. The 2017 Global Burden of Disease Study showed that cardiovascular and cerebrovascular diseases remain the leading cause of death (1). From 2007 to 2017, the death number of cardiovascular and cerebrovascular diseases worldwide increased by 21.1%. In 2015, the number of people who died of heart disease in the United States was around 630,000, accounting for a quarter of the total death toll (2). In recent years, China’s air pollution levels have risen abnormally with the booming economy. There are also many research indicate that air pollution has an impact on cardiovascular and cerebrovascular diseases (3–4).

With the rapid economic growth, China is inevitably faced with environmental pollution caused by the continuous increase in coal use, automobile exhaust emissions, power generation emissions and construction dust (5). Shenyang, a hub of modern manufacturing base and integrated transportation, is the capital city of Liaoning Province in China, which total area is 12,948 square kilometers. The resident population of 2015 is 8,291,100. It is located in northeast China and central Liaoning Province. It is mainly plain, and the mountains and hills are concentrated in the southeast. It is cold in winter with high frequency and intensity of temperature inversion, and it is easy to form haze, which is not conducive to the diffusion of pollutants. In addition, Shenyang is one of heavy industry cities in China, and the exhaust gas emitted by factories seriously affects the air pollution quality in Shenyang, which has potential adverse effects on public health (6).

Under these conditions, this research established the hypothesis that 6 types of air pollutants in Shenyang would cause adverse cardiovascular and cerebrovascular diseases admission, then carried out verification analysis. We established a generalized additive model and a distribution lag model to evaluate the short-term impact of air pollution on the admission rate of cardiovascular and cerebrovascular diseases in Shenyang from 2014 to 2017.

## Materials and Methods

We collected the daily average concentration data of air pollutants in Shenyang, Liaoning, China from Jan 1, 2014 to Dec 31, 2017, included the 24-hour average concentration of PM_2.5_, PM_10_, SO_2_, NO_2_ and CO, and the maximum 8-hour moving average concentration of O_3_, from Shenyang Environmental Monitoring Central Station. The average daily temperature, relative humidity and other meteorological data of the same period, from the environmental protection bureau of Shenyang, China were collected as well.

The admission data of this study were obtained from the daily admission of patients from the Central Hospital of Shenyang Medical College and Shenzhou Hospital of Shenyang Medical College, Liaoning, China from Jan 1, 2014 to Dec 31, 2017. Daily admission visits to the cardiovascular and cerebrovascular system included patients diagnosed firstly and those admitted previously in all departments. The admission rate data was recorded according to ICD-10. The medical information collected mainly includes the discharge department, age, gender, admission time, discharge time, outpatient (emergency) diagnosis, disease code, etc., recorded by the clinician into the computer system.

This research was approved by the Ethics Committee of Shenyang Medical College (Shenyang, China). All patients signed the informed consent. These data including daily data on cardiovascular and cerebrovascular disease admissions, major atmospheric pollutants, and meteorological conditions, which have obvious time-varying trends, are linked by date. Therefore, time series method can be selected to analyze the relationship between air pollution and admission of cardiovascular and cerebrovascular diseases. The modeling method refers to Chen’s research on the relationship between air pollution and hospital admission in Shanghai (7). Compared with the total population of Shenyang residents, the daily number of residents admitted to the hospital due to cardiovascular and cerebrovascular diseases is a very small probability event, which is nonlinear. Its statistical distribution is similar to the Poisson distribution. So Poisson regression generalized additive model (GAM) was chosen.

The most critical aspect of time series analysis is how to determine the best model that is consistent with the health of the city’s residents. The explanatory variables and response variables of time series data are measured from different time points, and the same variable has autocorrelation at different time points. In order to remove the sequence correlation of the number of daily admissions at each time point, the following modeling strategies were adopted in this study:

1. In order to adjust the long-term trend and seasonal changes of residents’ daily admissions, the natural three-time smooth spline function is introduced into the GAM model to control the sequence correlation and nonlinear trend of daily admissions. The annual degree of freedom (df) is 7.
2. Introduce the “Day of Week” (DOW) into the generalized additive model as a dummy variable, excluding the natural fluctuations of the number of daily admissions within a week.
3. The variables of daily average temperature, relative humidity are introduced into the generalized additive model to adjust the confounding effect of meteorological factors. Since the relationship between meteorological factors and the number of daily admissions are generally nonlinear, the natural smoothing spline function is chosen to adjust their mixing effects. The daily average temperature has a df of 6, and the relative humidity df is 3 to eliminate the potential nonlinearity and hysteresis confusion effects of weather conditions.

Considering that there may be a lag effect of atmospheric pollutants on residents’ health, which may last for many days, our study will introduce GAM to explore the potential delay and cumulative effects of pollutants using single-day lag (lag0∼Lag3) and moving average exposure lag (lag01 to lag03). We also analyze different gender data. Differences in the effects of air pollution on admission to cardiovascular and cerebrovascular diseases in different groups were examined by calculating a 95% confidence interval (95% CI).

Based on the above strategies, this study establishes the basic model as follows:

$$\begin{array}{l} \text{logE}\left({\text{Y}}_{\text{t}}\right)=\alpha +\beta {\text{Z}}_{\text{t}}+\text{DOW}+\text{ns}\;\left(\text{time},7*5\right)+\text{ns}\\ \left(\text{temperature},6\right)+\text{ns}\;\left(\text{relative}\;\text{humidity},3\right) \end{array}$$

Where: E(Yt)—the daily number of hospital admissions observed daily; α—intercept; Zt—Atmospheric pollutant concentration at t day; β—Regression coefficient of the relationship between air pollutants and hospitalized patients; DOW—day of week; ns—the natural smoothing spline function, df — the degree of freedom .

Statistical analysis was performed using the MGCV software package in R language software version 3.5.1. Statistical tests were bilateral and (*P*< 0.05) was considered statistically significant. Results are expressed as a percent change (mean and 95% confidence intervals) of daily outpatient visits for cardiovascular and cerebrovascular diseases per 10 μg/m^3^ increase in NO_2_, O_3_, PM_10_, PM_2.5_, SO_2_ concentration, and per 1 mg/m^3^ increase in CO concentration.

## Results

Table 1 summarizes the basic descriptive statistics of hospital admissions of cardiovascular and cerebrovascular diseases, air quality and meteorological data. From Jan 1, 2014 to Dec 31, 2017, overall 157,144 patients were admitted to the hospital with cardiovascular and cerebrovascular diseases, including 7,700 males and 80,143 females. The average daily number of hospital admissions was 107. Correlation analysis between different atmospheric pollutants and meteorological factors showed that there was a significant correlation between other atmospheric pollutants and meteorological factors except relative humidity and PM_2.5_, relative humidity and NO_2_, relative humidity and (*P*<0.05) (Table 2). Table 3 shows the results of the single lag model (lag0 to lag3) and the cumulative exposure model (lag01 to lag03) with gender. At lag=0, it is generally a positive and statistically significant association between NO_2_, O_3_, PM_10_ and cardiovascular and cerebrovascular diseases was observed in the general population. Under each pollution model, the overall effect intensity of the three pollutants is NO_2_>O_3_>PM_10_. Gender specific analysis showed that when lag=0, female had a slight influence on admission of cardiovascular and cerebrovascular diseases in terms of CO, NO_2_, PM_2.5_, PM_10_ and O_3_. Male were affected by NO_2_, PM_10_ and O_3_ in lag3 day. We did not observe a strong correlation between PM_2.5_, CO, SO_2_ and the total population admission, especially SO_2_. Therefore, in the subsequent analysis, we did not continue to analyze the relationship between PM_2.5_, CO, SO_2_ and the admission of cardiovascular and cerebrovascular diseases.

**Table 1:** Descriptive analysis of daily admission of cardiovascular and cerebrovascular diseases, daily concentration of atmospheric pollutants, and daily weather conditions

***Variable***	***Mean***	***SD***	***Min***	***P25***	***P50***	***P75***	***Max***
PM_2.5_(μg/m^3^)	62.67	51.41	4	32	48	79	885.0
PM_10_(μg/m^3^)	105.12	71.82	9	61.5	89	129	1331
SO_2_(μg/m^3^)	57.95	62.12	4	17	32	78	392
NO_2_(μg/m^3^)	45.10	17.29	15	32	43	54	140
O_3_(μg/m^3^)	93.21	48.65	11	54	86	126	283
CO (mg/m^3^)	0.996	0.47	0.3	0.7	0.9	1.2	3.3
Daily admission(person)	107.60	46.34	0	66	108	139	253
Sex							
Male (person)	52.72	23.58	0	32	52	68	136
Female (person)	54.87	24.31	0	34	54	72	142
Age							
0–30(years)	0.56	0.82	0	0	0	1	5
31–60(years)	37.97	18.10	0	22	38	51	99
>60(years)	69.07	29.99	0	44	67	89	168
Relative Humidity (%)	2.41	0.95	0	1.7	2.21	3.00	6.00
Temperature(°C)	9.17	12.87	−20.5	−2.39	11.00	20.87	31.9

**Table 2:** Spearman correlation data of atmospheric pollutant concentration and meteorological data in Shenyang City from 2014 to 2017

***Variables***	***PM_10_***	***PM_2.5_***	***SO_2_***	***NO_2_***	***CO***	***O_3_***	***temperature***	***relative humidity***
PM_10_	1	0.91^**^	0.67^**^	0.69^**^	0.72^**^	−0.20^**^	−0.37^**^	−0.17^**^
PM_2.5_	-	1	0.70^**^	0.75^**^	0.82^**^	−0.25^**^	−0.39^**^	0.03
SO_2_	-	-	1	0.70^**^	0.63^**^	−0.56^**^	−0.76^**^	−0.22^**^
NO_2_	-	-	-	1	0.73^**^	−0.37^**^	−0.44^**^	−0.02
CO	-	-	-	-	1	−0.19^**^	−0.24^**^	0.22^**^
O_3_	-	-	-	-	-	1	0.81^**^	0.01
Temperature	-	-	-	-	-	-	1	0.30^**^
Relative Humidity	-	-	-	-	-	-	-	1

**Table 3:** Percent change (mean and 95% confidence intervals) of daily outpatient visits for cardiovascular and cerebrovascular diseases diseases per 10 μg/m^3^ increase in NO_2_, O_3_, PM_10_, PM_2.5_, SO_2_ concentration, per 1 mg/m^3^ increase in CO concentration at different lag days in different sex model

***Pollutant***	***Lag days***	***All***	***Male***	***Female***
CO	0	0.957(− 0.209,2.138)	−0.025(−1.920,1.908)	1.832(0.308,3.379)
CO	1	−0.949(−2.317,0.439)	−1.191(−3.271,0.934)	−0.763(−2.680,1.193)
CO	2	−3.289(−4.991, −1.557)	−4.347(−7.205, −1.400)	−2.535(−4.785, −0.231)
CO	3	−1.034(−2.366,0.317)	−0.828(−2.762,1.144)	−1.304(−3.257,0.687)
CO	0–1	−0.697(−2.357,0.991)	−1.946(−4.769,0.960)	0.363(−1.778,2.551)
CO	0–2	−2.420(−4.746, −0.037)	−3.777(−7.899,0.529)	−1.273(−4.270,1.818)
CO	0–3	−3.744(−6.811, −0.575)	−4.850(−10.303,0.935)	−2.689(−6.713,1.507)
NO_2_	0	1.508(1.159,1.856)	0.93(0.428,1.433)	2.027(1.544,2.51)
NO_2_	1	0.51(0.165,0.855)	0.211(−0.286,0.708)	0.749(0.27,1.229)
NO_2_	2	−0.67(−1.012, −0.327)	−0.872(−1.365, −0.379)	−0.498(−0.974, −0.022)
NO_2_	3	−0.535(−0.878, −0.192)	−0.442(−0.935,0.051)	−0.627(−1.104, −0.15)
NO_2_	0–1	1.49(1.083,1.898)	0.942(0.356,1.528)	1.986(1.42,2.551)
NO_2_	0–2	1.522(1.057,1.988)	1.024(0.356,1.692)	1.998(1.35,2.646)
NO_2_	0–3	1.492(0.976,2.008)	1.005(0.264,1.746)	1.967(1.248,2.686)
O_3_	0	0.355(0.159,0.55)	0.297(0.02,0.575)	0.423(0.148,0.698)
O_3_	1	0.217(0.028,0.405)	0.307(0.039,0.575)	0.165(−0.101,0.43)
O_3_	2	0.252(0.068,0.436)	0.345(0.084,0.606)	0.159(−0.1,0.418)
O_3_	3	0.547(0.367,0.728)	0.562(0.306,0.817)	0.534(0.28,0.787)
O_3_	0–1	0.307(0.126,0.488)	0.24(−0.017,0.497)	0.371(0.117,0.625)
O_3_	0–2	0.165(−0.007,0.337)	0.135(−0.109,0.379)	0.189(−0.053,0.431)
O_3_	0–3	0.13(−0.037,0.298)	0.116(−0.122,0.354)	0.145(−0.091,0.381)
PM_10_	0	0.084(0.003,0.165)	0.039(−0.079,0.157)	0.122(0.011,0.233)
PM_10_	1	−0.321(−0.398, −0.243)	−0.3(−0.412, −0.189)	−0.349(−0.456, −0.241)
PM_10_	2	−0.174(−0.252, −0.096)	−0.176(−0.289, −0.063)	−0.178(−0.285, −0.071)
PM_10_	3	0.133(0.061,0.205)	0.169(0.065,0.273)	0.098(−0.002,0.198)
PM_10_	0–1	−0.103(−0.201, −0.005)	−0.147(−0.289, −0.004)	−0.065(−0.2,0.071)
PM_10_	0–2	−0.246(−0.359, −0.134)	−0.278(−0.441, −0.116)	−0.215(−0.371, −0.06)
PM_10_	0–3	−0.31(−0.435, −0.186)	−0.318(−0.497, −0.139)	−0.31(−0.482, −0.138)
PM_2.5_	0	0.11(−0.004,0.225)	0.011(−0.156,0.178)	0.198(0.042,0.355)
PM_2.5_	1	−0.205(−0.313, −0.097)	−0.23(−0.387, −0.073)	−0.191(−0.341, −0.041)
PM_2.5_	2	−0.125(−0.233, −0.017)	−0.141(−0.297,0.015)	−0.117(−0.265,0.032)
PM_2.5_	3	0.07(−0.032,0.173)	0.134(−0.013,0.282)	0.009(−0.134,0.151)
PM_2.5_	0–1	−0.052(−0.185,0.08)	−0.132(−0.323,0.06)	0.022(−0.16,0.204)
PM_2.5_	0–2	−0.163(−0.311, −0.015)	−0.222(−0.436, −0.007)	−0.103(−0.308,0.101)
PM_2.5_	0–3	−0.228(−0.39, −0.066)	−0.256(−0.49, −0.022)	−0.205(−0.429,0.019)
SO_2_	0	0.033(−0.104,0.171)	−0.015(−0.214,0.183)	0.078(−0.113,0.268)
SO_2_	1	−0.024(−0.16,0.111)	−0.007(−0.202,0.189)	−0.035(−0.223,0.152)
SO_2_	2	−0.062(−0.198,0.073)	−0.12(−0.316,0.076)	−0.029(−0.216,0.158)
SO_2_	3	0.027(−0.107,0.16)	0.091(−0.102,0.284)	−0.046(−0.232,0.139)
SO_2_	0–1	−0.028(−0.186,0.129)	−0.065(−0.292,0.163)	−0.009(−0.227,0.209)
SO_2_	0–2	−0.064(−0.24,0.111)	−0.072(−0.326,0.181)	−0.08(−0.323,0.164)
SO_2_	0–3	−0.091(−0.281,0.098)	−0.064(−0.337,0.21)	−0.141(−0.404,0.122)

Table 4 provides the results of the dual-pollutant models with lag0 day. After adjustment, we observed that the value still showed a trend of NO_2_>O_3_>PM_10_, which was consistent with the results in Table 3. After adjustment, the influences of the dual-pollutant models of O_3_ and NO_2_ still have a positive correlation. After adjusting PM_10_ and NO_2_, the impact of O_3_ on hospital admission decreased by 0.025% and 0.069%. Compared with the NO_2_ single pollutant model, the dual pollution model of NO_2_ and PM_10_ increased slightly by 0.409%, while the dual pollutant model of NO_2_ and O_3_ decreased slightly by 0.039%. PM_10_ had no statistical significance after adjusting O_3_. The dual model of PM_10_ and NO_2_ showed a negative correlation.

**Table 4:** Percent increase in number of daily outpatient visits for cardiovascular and cerebrovascular diseases associated with a 10μg/m^3^(PM_10_, O_3_, and NO_2_) increase in air pollutant concentrations in dual-pollutant models

***Air pollutant models***		***Estimates***
PM_10_	-	0.084(0.003,0.165)
	+O_3_	0.061(−0.021,0.143)
	+NO_2_	−0.169(−0.272, −0.067)
O_3_	-	0.355(0.159,0.55)
	+PM_10_	0.33(0.131,0.528)
	+NO_2_	0.286(0.09,0.481)
NO_2_	-	1.508(1.159,1.856)
	+PM_10_	1.917(1.49,2.344)
	+O_3_	1.469(1.12,1.818)

**Fig. 1:**
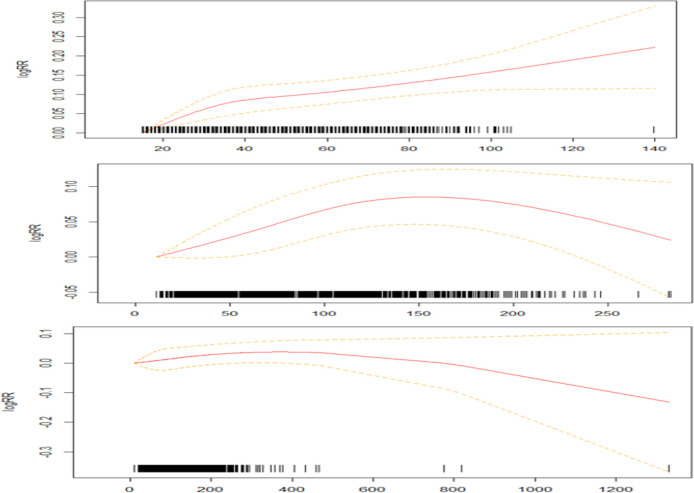
The exposure-response curve of pollutants concentrations and hospital admissions on cardiovascular and cerebrovascular diseases. X-axis is the pollutants’ concentrations (μg/m ^3^ for NO_2_, O_3_ and PM_10_) at concurrent days. The Y-axis is the log relative risk of outpatient visits for cardiovascular and cerebrovascular diseases with per 10 μg/m^3^ increase in pollutant concentration. The estimated mean percentages of change in daily outpatient visits for cardiovascular and cerebrovascular diseases are shown by the solid lines, and the dotted lines represent twice the point-wise standard errors, which mean 95% confidence intervals

Figure 1 shows the relationship between NO_2_, O_3_, PM_10_ in lag0 day and the hospital admission of cardiovascular and cerebrovascular diseases. From these curves, the correlation of NO_2_ seems to be positive, which is a relatively high linear relationship in the range of concentrations 20–40 μg/m^3^.

When the concentration is more than 40 μg/m^3^, the slope of relative risk correlation decreases slightly, but it still presents a basic linear relationship. The overall trend of O_3_ and PM_10_ is going up and down later. O_3_ exhibits a positive correlation at 0–150 μg/m^3^ and a negative correlation at concentrations greater than 150 μg/m^3^. PM_10_ concentration showed a weak positive correlation at 0–400 μg/m^3^, and a weak linear downward trend at 400–800μg/m^3^, and a strong downward trend when the concentration was greater than 800 μg/m^3^.

## Discussion

This research confirmed that air pollution in Shenyang has a certain impact on residents’ admission of cardiovascular and cerebrovascular diseases. Among them, the effect is NO_2_>O_3_>PM_10_. At present, most of the studies on air pollution are focus on tropical, subtropical, and sub-cold zones. There are few studies on air pollution and the admission to hospital of residents’ cardiovascular and cerebrovascular diseases in temperate zones, especially in northeast China. Therefore, this study is a supplement for the assessment of the impact of air pollution on residents’ admission to hospital with cardiovascular and cerebrovascular diseases in northeast China. Furthermore, most of the current studies on the correlation between air pollutants and cardiovascular and cerebrovascular diseases mainly focus on 2 or 3 pollutants, while this study analyzed 6 types of air pollutants, which is very rare.

The air pollution in Shenyang (including NO_2_, O_3_, PM_10_) has a certain impact on the hospitalization of residents with cardiovascular and cerebrovascular diseases in the general population, verifying the correlation between the air pollution and cardiovascular and cerebrovascular diseases. In this research, it was observed that NO_2_, O_3_, and PM_10_ showed a significant association with admission on lag0 day generally, and the association in the cumulative exposure model was still positive. The results show that residents in Shenyang are susceptible to these pollutants and suffer acute reactions in a short period, which is consistent with previous research results (8–9).

This study confirmed that the interaction of pollutants could change the risk of admission of cardiovascular and cerebrovascular diseases. In the dual-pollutant models, the interaction risk of NO_2_ and PM_10_ is increased, so the interaction between NO_2_ and PM_10_ has a greater impact on the admission of cardiovascular and cerebrovascular diseases. In the remaining dual-pollutant models, the risk of admission was slightly reduced compared with that of single pollution. It can be seen that the interaction between atmospheric pollutants affects the effect of single pollutants on the health of cardiovascular and cerebrovascular diseases, which may be due to the collinearity of pollutants, and many unknown potential factors in the environment have enhanced or weakened effects on the interaction of pollutants. Moreover, human and animal experiments have shown that exposure to a variety of mixed pollutants causes physiological changes in the body, such as cardiovascular and pulmonary changes, than exposure to a single pollutant, suggesting a synergistic effect (10). However, experimental research could achieve observation that is more accurate and assessment of the health effects of atmospheric pollutant interactions by controlling experimental conditions. This is somewhat different from the outcome of exposure to the real atmospheric environment. The results of this study and other related studies at home and abroad indicate that there is an interaction between atmospheric pollutants (11–12).

This study is partially inconsistent with the expected assumptions. We found that SO_2_, CO and PM_2.5_ have no independent contribution to the admission of cardiovascular and cerebrovascular diseases for the total population, which is different from previous research results (13–15). It may be that the annual average concentration of SO_2_ and the maximum daily average concentration of CO did not exceed the limits required by national health standards, so the pollution is not serious. Differences in study methods, long-term and short-term exposure, or other conditions (such as air pollution levels, ethnicity, and the health of residents) may also account for the study results. A study based on the ability of particles to oxidize ascorbic acid (OPAA) and glutathione (OPGSH) to evaluate the oxidation potential of particles and daily mortality and hospitalization found that there are no evidence to support the hypothesis that short-term exposure to particulate PM_2.5_ is associated with adverse cardiovascular disease effects (16). Therefore, it is necessary to carry out further research in the future to confirm accurately the role of PM_2.5_ in causing disease progression.

This study suggests that women are more susceptible to the immediate effects of admission to hospital induced by atmospheric pollutants, while men are more susceptible to the delayed effects of admission caused by pollutants. CO, NO_2_, O_3_, PM_10_, PM_2.5_ had a weak effect on the admission of female patients cardiovascular and cerebrovascular diseases on the day of exposure. Some physiological and social factors studies can explain this phenomenon. First, the diameter of the respiratory tract of women is smaller on anatomically, so women show higher airway response and particulate deposition effect compared with men (17). Secondly, male smokers outnumber females in most parts of China. A study showed that the influence of air pollutants on non-smokers is heavier than that of smokers (18). Thirdly, differences in gender could cause differences in the occupations that can be performed and the level of education they receive, and thus differences in exposure (19).

The concentration-response curve of this study can provide a reference for the risk assessment of atmospheric pollutants and the formulation of public policies. The shape of exposure-response shows that NO_2_ seems to be approximately linearly related to the rate of cardiovascular and cerebrovascular visits, and the trend increases with the concentration. O_3_ and PM_10_ are positively correlated with admission at low concentration levels, and the curves showed a downward trend after the concentrations reached a certain threshold. The reason may be that susceptible people may have been admitted to the hospital before the concentration of air pollutants reaches a relatively high level.

The study has some limitations. Firstly, like other time series studies, the daily average air pollutant concentrations at the stations are used to represent for the real population exposure. This might lead to measurement errors, because of personal exposure depends on many situations, such as indoor and outdoor activities, location of the residence, occupational exposure, and so on. Secondly, the history of hospital visits only comes from two hospitals in Shenyang China, which may affect the prevalence of epidemiological results. Thirdly, the study only involved four years, because the Shenyang hospital did not conduct electronic management of admission data before 2014. Fourthly, other possible confounding factors, such as BMI, education and income, smoking, physical activity, and medical history, were not included in this study, which may also have a potential impact on the association between air pollutants and admission to hospital for cardiovascular and cerebrovascular diseases.

## Conclusion

Short-term exposure to environmental NO_2_, PM_10_, and O_3_ increases the risk of admission to cardiovascular and cerebrovascular diseases. At present, it is urgent to carry out large-scale prospective cohort study to reflect accurately the relationship between the health effects of air pollutants on human body.

## Ethical considerations

Ethical issues (Including plagiarism, informed consent, misconduct, data fabrication and/or falsification, double publication and/or submission, redundancy, etc.) have been completely observed by the authors.
